# RUNX3 mediates keloid fibroblast proliferation through deacetylation of EZH2 by SIRT1

**DOI:** 10.1186/s12860-022-00451-4

**Published:** 2022-12-07

**Authors:** Hanye Liu, Guanghai Yan, Li Li, Dandan Wang, Yu Wang, Shan Jin, Zhehu Jin, Liangchang Li, Lianhua Zhu

**Affiliations:** 1 Jilin Key Laboratory for Immune and Targeting Research On Common Allergic Diseases, Yanbian University, Yanji, 133000 People’s Republic of China; 2 Department of Anatomy, Histology and Embryology, Medical College, Yanbian University, No. 977 Gongyuan Road, Yanji, 133002 People’s Republic of China; 3 Department of Dermatology, Yanbian University Hospital, Yanji, 133002 People’s Republic of China

**Keywords:** Keloid, Abnormal proliferation, Cell cycle, SIRT1/EZH2/RUNX3 axis

## Abstract

**Background:**

Keloid is a benign proliferative fibrous disease featured by excessive fibroblast proliferation after skin injury. However, the mechanism of abnormal cell proliferation is still unclear. Herein, we investigated the mechanism of abnormal proliferation in keloids involving Sirtuin 1(SIRT1)/ Zeste Homolog 2 (EZH2)/ Runt-related transcription factor 3 (RUNX3).

**Methods:**

HE staining was used to observe the histopathological changes. Western blot was performed to detect SIRT1/EZH2/RUNX3 and cell cycle related proteins. RT-PCR detected *EZH2* mRNA. After knockdown of EZH2 or overexpression of RUNX3, cell proliferation and cell cycle was analyzed. Immunoprecipitation was used to detect acetylated EZH2.

**Results:**

The results showed that overexpression of RUNX3 inhibited cell proliferation and arrested cell cycle at G1/S phase, whereas inhibition of SIRT1 promoted cell proliferation and G1/S phase of the cell cycle. Knockdown of EZH2 promoted the expression of RUNX3, inhibited cell proliferation and shortened the progression of G1 to S phase. Simultaneous knockdown of EZH2 and inhibition of SIRT1 reversed these effects. Inhibition of SIRT1 increased its protein stability by increasing EZH2 acetylation, thereby reducing the expression of RUNX3 and promoting cell proliferation.

**Conclusions:**

Conclusively, the SIRT1/EZH2/RUNX3 axis may be an important pathway in the regulation of abnormal proliferation in keloids.

**Supplementary Information:**

The online version contains supplementary material available at 10.1186/s12860-022-00451-4.

## Background

Keloid is a benign but locally aggressive and recurrent fibroproliferative disease with clinical features such as rapid growth, infiltration into normal tissue and easy recurrence after resection. It is a skin lesion that heals abnormally after skin tissue damage [1, 2]. The pathogenesis of keloid is complex. It is reported that abnormal proliferation of skin fibroblasts leads to increased cellular collagen secretion, which in turn promotes connective tissue proliferation or deformation and ultimately leads to scar formation [3]. Keloid has seriously affected the physical and mental health of patients. Surgery, drugs, and laser are the main methods for the clinical treatment of keloid. However, the therapeutic effect is poor and the recurrence rate of keloid is high [4]. Studies [5, 6] have shown that keloids can be improved by promoting fibroblast apoptosis and inhibiting fibroblast proliferation. The abnormal proliferation and infiltration of keloid fibroblasts resembles the characteristics of tumor cells, but the mechanism of their abnormal proliferation remains unclear.

The Runt-related transcription factor (RUNX) family includes context-dependent transcriptional regulators in humans, including RUNX1, RUNX2, and RUNX3.The RUNX family has a highly conserved Runt homology domain that mediates site-specific DNA binding and interactions with various proteins [7]. RUNX1 is essential for hematopoiesis. RUNX2 is involved in osteogenesis during bone formation. RUNX3 is expressed in epithelial, neuronal, and hematopoietic stem cells, and participates in the transforming growth factor beta (TGF-β) signaling pathway [8], in which its function is to regulate lineage differentiation of hematopoietic and neuronal progenitors [9]. RUNX3 has been demonstrated to function as a tumor suppressor in many types of tumors [10–12]. It can have a therapeutic effect on keloid by increasing the sensitivity of keloid fibroblasts to PaPDT [13]. Studies have shown that RUNX3 promoter hyper-methylation is a key factor in tumorigenesis and proliferation [14, 15]. Meanwhile, downregulation of Zeste Homolog 2 (EZH2) can reduce the methylation level of RUNX3, and silencing EZH2 can inhibit the re-methylation of tumor cells [16]. EZH2 is the enzymatic catalytic subunit of polycomb repressive complex 2 and can regulate the expression of downstream target genes through trimethylation of Lys-27 in histone 3 (H3K27me3). In addition to H3K27me3, EZH2 can regulate gene expression in other ways [17]. EZH2 expression is higher in many malignant tumors than in normal tissues and its high expression is closely related to epithelial-mesenchymal transition [18]. It has been reported that post-translational modification of EZH2 plays an important role in regulating downstream target gene silencing and function [19]. However, the post-translational modification of EZH2 has rarely been reported in keloids.

Sirtuin1 (SIRT1) is a class III histone deacetylase and an NAD^+^-dependent enzyme that is closely related to gene regulation, genome stability maintenance, apoptosis, autophagy, proliferation, senescence, and tumorigenesis [20]. Deacetylation of EZH2 by SIRT1 in lung adenocarcinoma can affect its stability thereby inhibiting cell migration and invasion [21]. SIRT1 upregulation has been demonstrated in some cancer cells, such as acute myelogenous leukemia and primary colon cancer, prostate cancer, melanoma and non-melanoma skin cancer [22, 23]. In keloids, SIRT1 can exacerbate cell death by affecting the SIRT3-SOD2-mROS-dependent autophagy pathway corresponding to 5-ALA-PDT [24]. The mechanism of cell proliferation involving SIRT1/EZH2/RUNX3 in benign keloids is still unclear.

Herein, we aim to investigate the role and mechanism of SIRT1/EZH2/RUNX3 in abnormal proliferation of benign keloids. Our findings may provide a theoretical basis for the treatment of keloid.

## Results

### Correlation of SIRT1/RUNX3/EZH2 expression in keloids

**Fig. 1:**
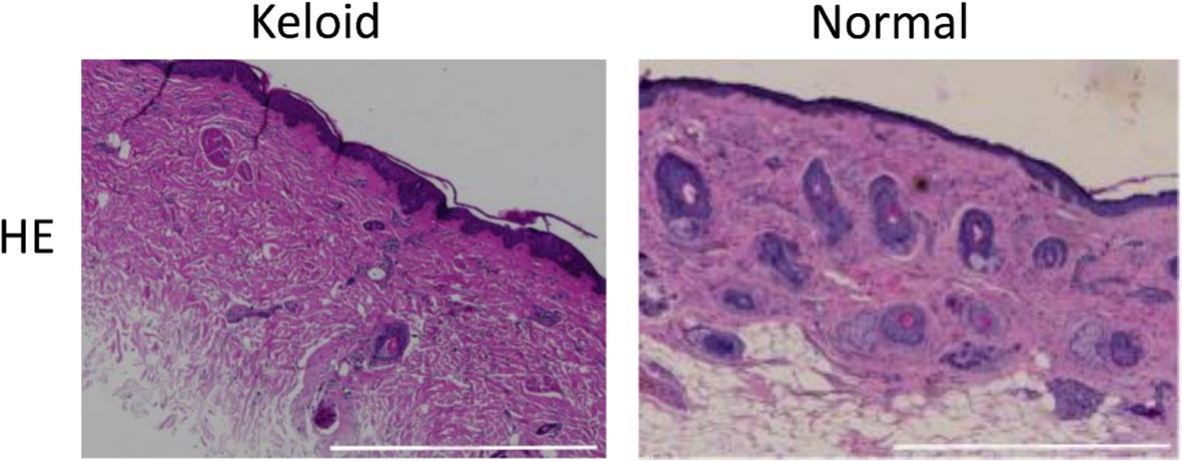
Keloid histopathology. HE staining; Scale bar, 1000 μm

HE staining analyzed the pathological changes. As shown in Fig. 1, in keloid tissues, the dermis layer was thickened and some fibroblasts were observed in the superficial dermis. Collagen fiber bundles were obviously thickened compared with the superficial dermis, and hyalinization was observed. Compared with normal skin, cell infiltration was obvious in the middle dermis; collagen fibers were irregularly arranged in the deep dermis; and the number of cells was less in keloid tissues.

**Fig. 2:**
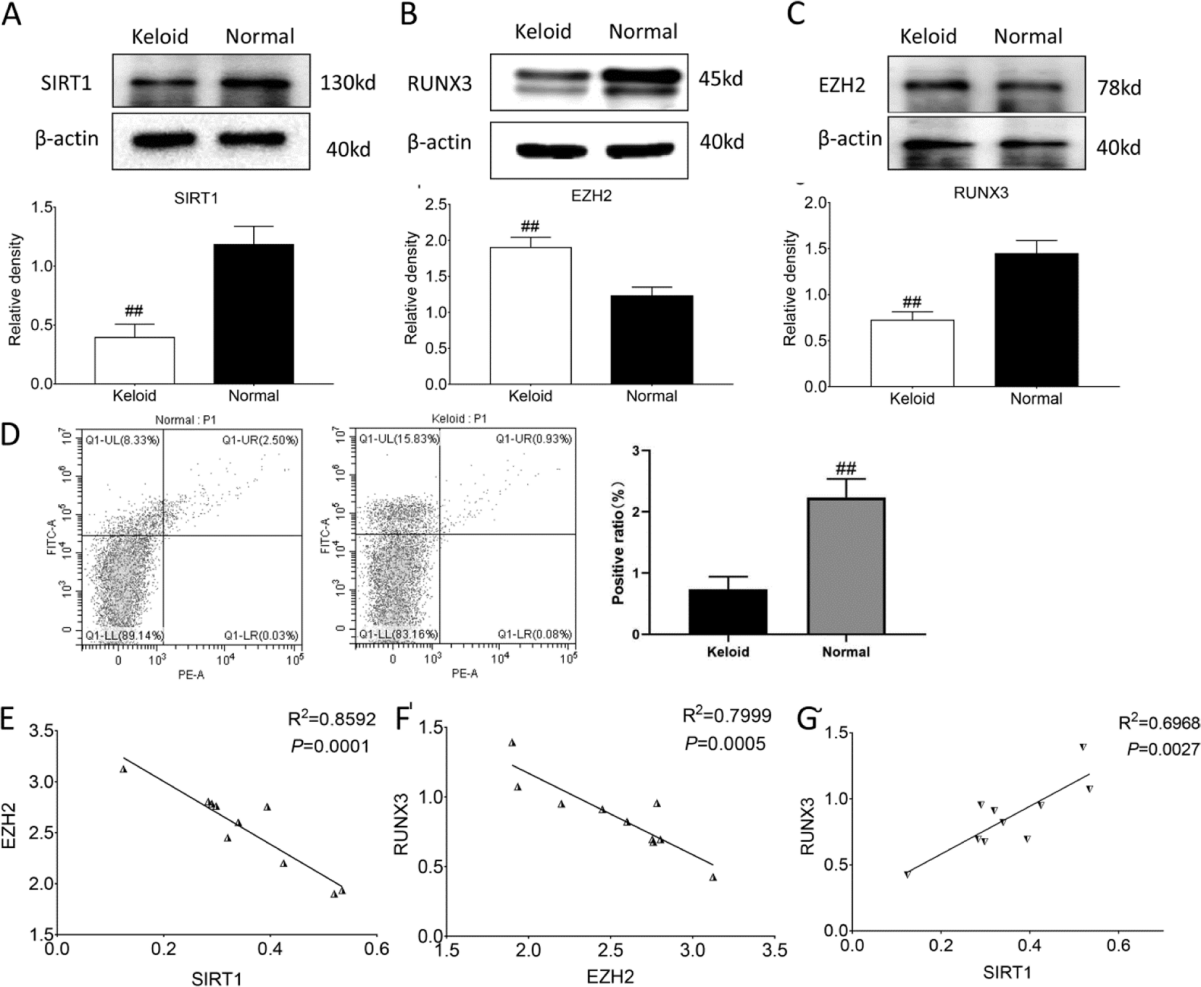
Analysis of SIRT1/RUNX3/EZH2 expression in keloids. Representative and quantitative Western blot results of SIRT1 (**A**), RUNX3 (**B**), and EZH2 (**C**); ##*P*˂0.01 vs Normal. Original blots are presented in Supplementary Fig. 1. **D** Flow cytometry analysis of RUNX3 expression in single cell suspensions of skin tissues with specific vimentin expression. The percentage in the upper right quadrant indicates RUNX3 expression in fibroblasts of skin tissues. ##*P*˂0.01 vs Normal. **E** Correlation analysis between SIRT1 and EZH2. **F** Correlation analysis between EZH2 and RUNX3. **G** Correlation analysis between SIRT1 and RUNX3

**Fig. 3:**
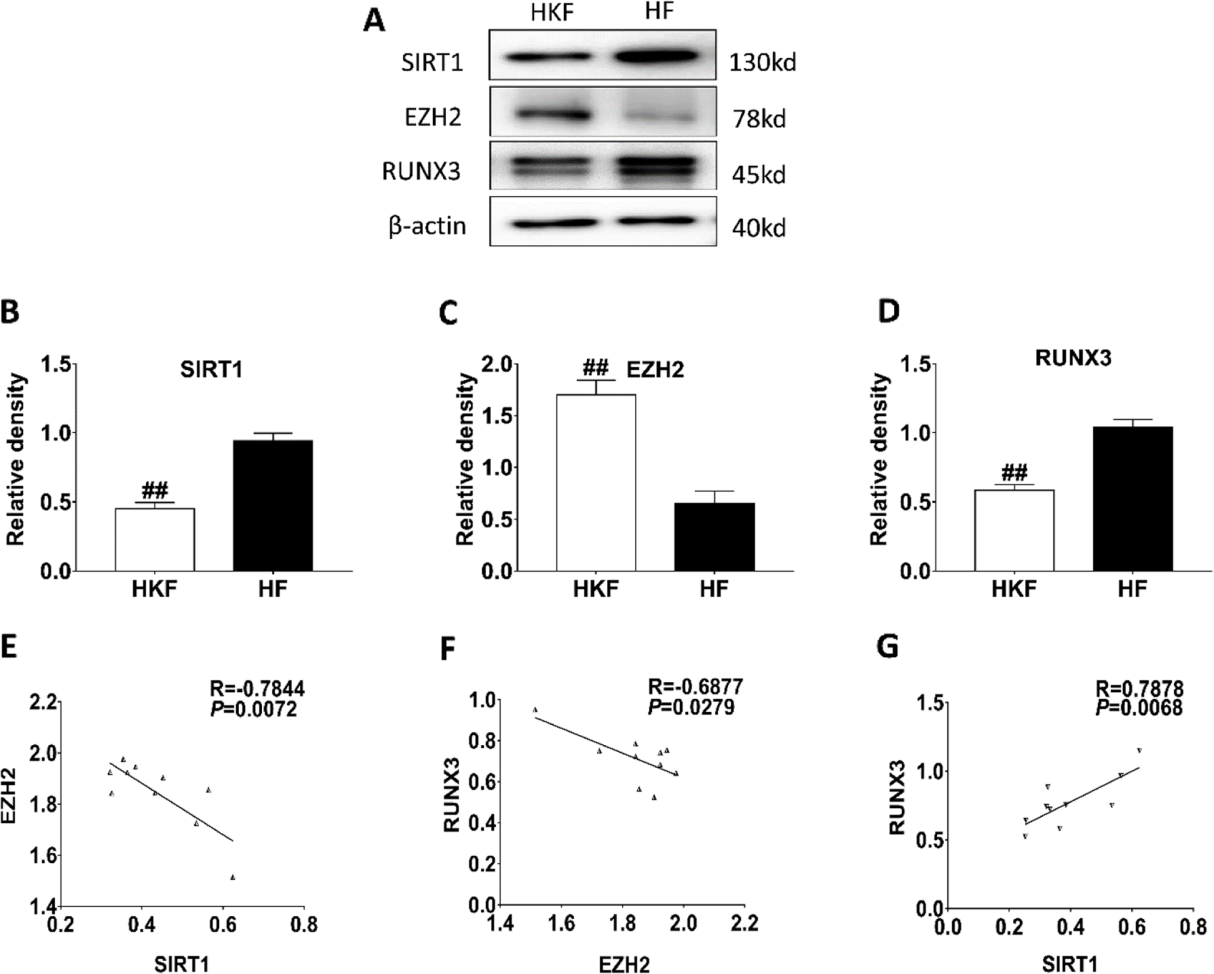
Analysis of SIRT1/RUNX3/EZH2 expression in Keloid fibroblast. (**A**) Representative Western blot results of SIRT1, EZH2, and RUNX3 in human keloid fibroblasts (HKF) and human fibroblasts (HF); Original blots are presented in Supplementary Fig. 2. Quantitative Western blot results of SIRT1 (**B**), EZH2 (**C**), and RUNX3 (**D**). ##*P*˂0.01 vs HF. (**E**) Correlation analysis between SIRT1 and EZH2; (**F**) Correlation analysis between EZH2 and RUNX3; (**G**) Correlation analysis between SIRT1 and RUNX3

The expression levels of SIRT1, EZH2 and RUNX3 in keloid specimens and their normal adjacent tissues were detected by Western blot. The results showed that the expression levels of SIRT1 (*P* < 0.05, Fig. 2A) and RUNX3 (*P* < 0.05, Fig. 2B) in keloid tissues were significantly lower than those in normal adjacent skin tissues. However, the expression level of EZH2 in keloid tissues was significantly higher than that in adjacent skin tissues (*P* < 0.05, Fig. 2C). To further verify the expression of RUNX3 in normal and Keloid skin tissues, we detected the co-expression of RUNX3 and vimentin in single cell suspensions of skin tissues by using flow cytometry. Consistently, we found that there was RUNX3 expression in cells specifically expressing vimentin and that RUNX3 expression in normal skin was significantly higher than in Keloid skin (Fig. 2D). In addition, the correlation analysis showed that there was negative regulatory relationship between SIRT1 and EZH2 (*P* < 0.05, Fig. 2E), and, between EZH2 and RUNX3 (*P* < 0.05, Fig. 2F). There was positive correlation between SIRT1 and RUNX3 (*P* < 0.05, Fig. 2G). Similar results of SIRT1, EZH2 and RUNX3 were also observed in keloid fibroblasts and normal fibroblasts (Fig. 3A-G).

### Effect of overexpression of RUNX3 on proliferation of keloid fibroblasts

**Fig. 4:**
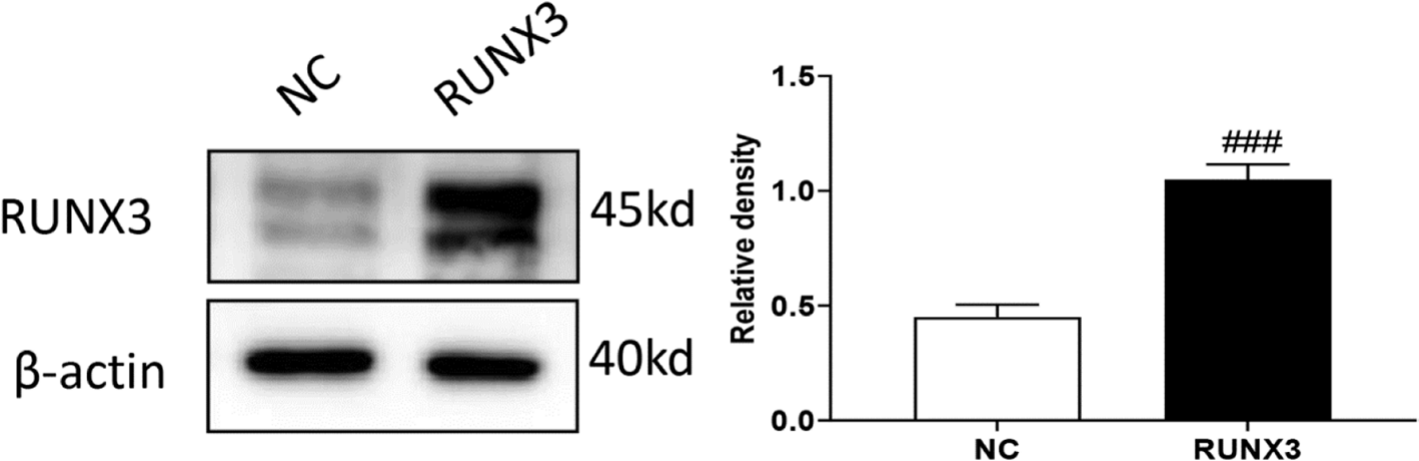
Overexpression of RUNX3. Representative and quantitative Western blot results of RUNX3. Original blots are presented in Supplementary Fig. 3. ###*P*˂0.001 vs NC

**Fig. 5:**
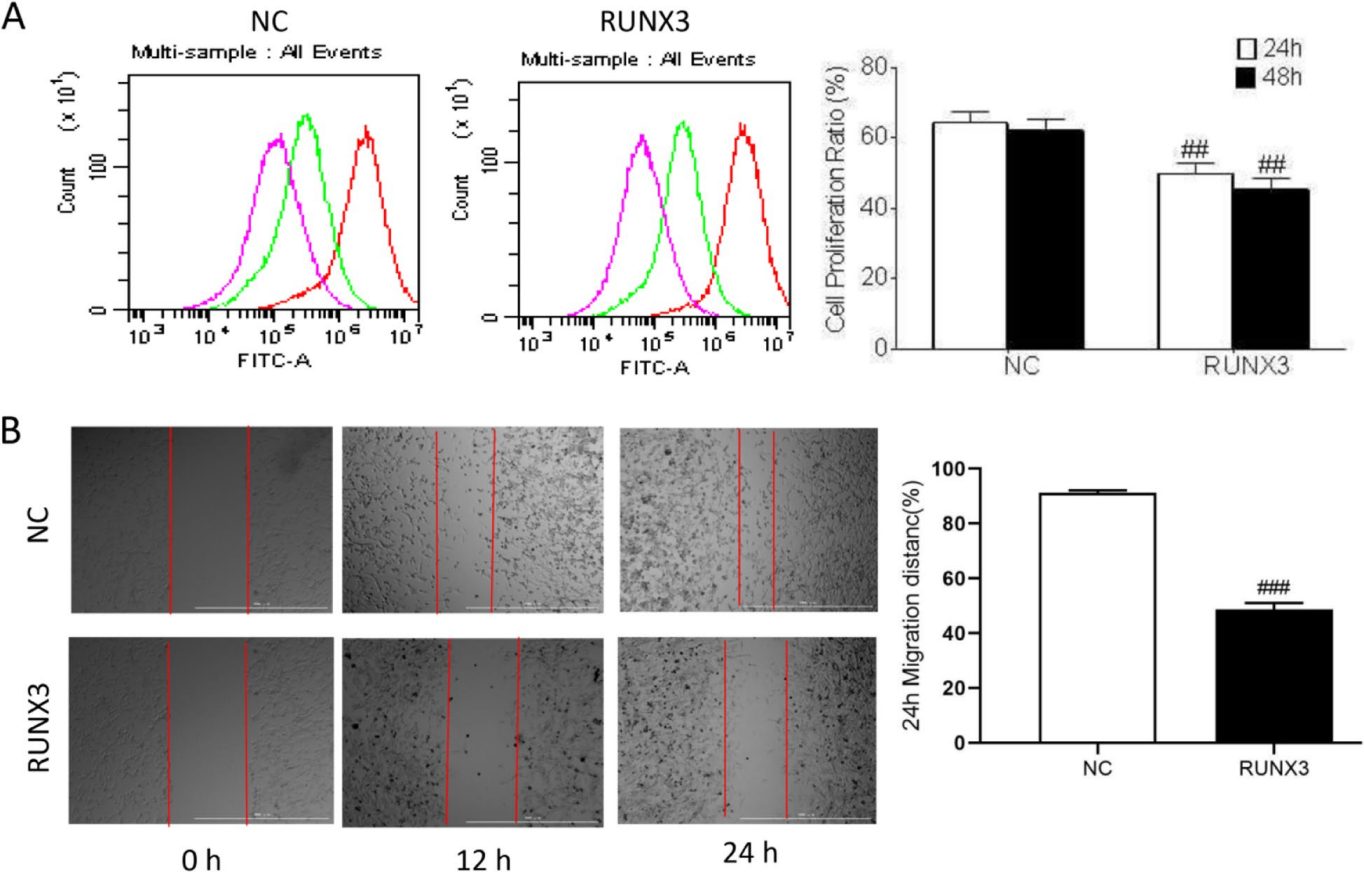
Effect of overexpression of RUNX3 on proliferation of human keloid fibroblasts. **A** Effect of overexpression of RUNX3 on cell proliferation as assessed by CFSE. Green line indicates proliferation at 24 h and red line represent proliferation at 48 h; ##*P*˂0.01 vs NC. **B** Cell scratch assay. Scale bar, 1000 μm

**Fig. 6:**
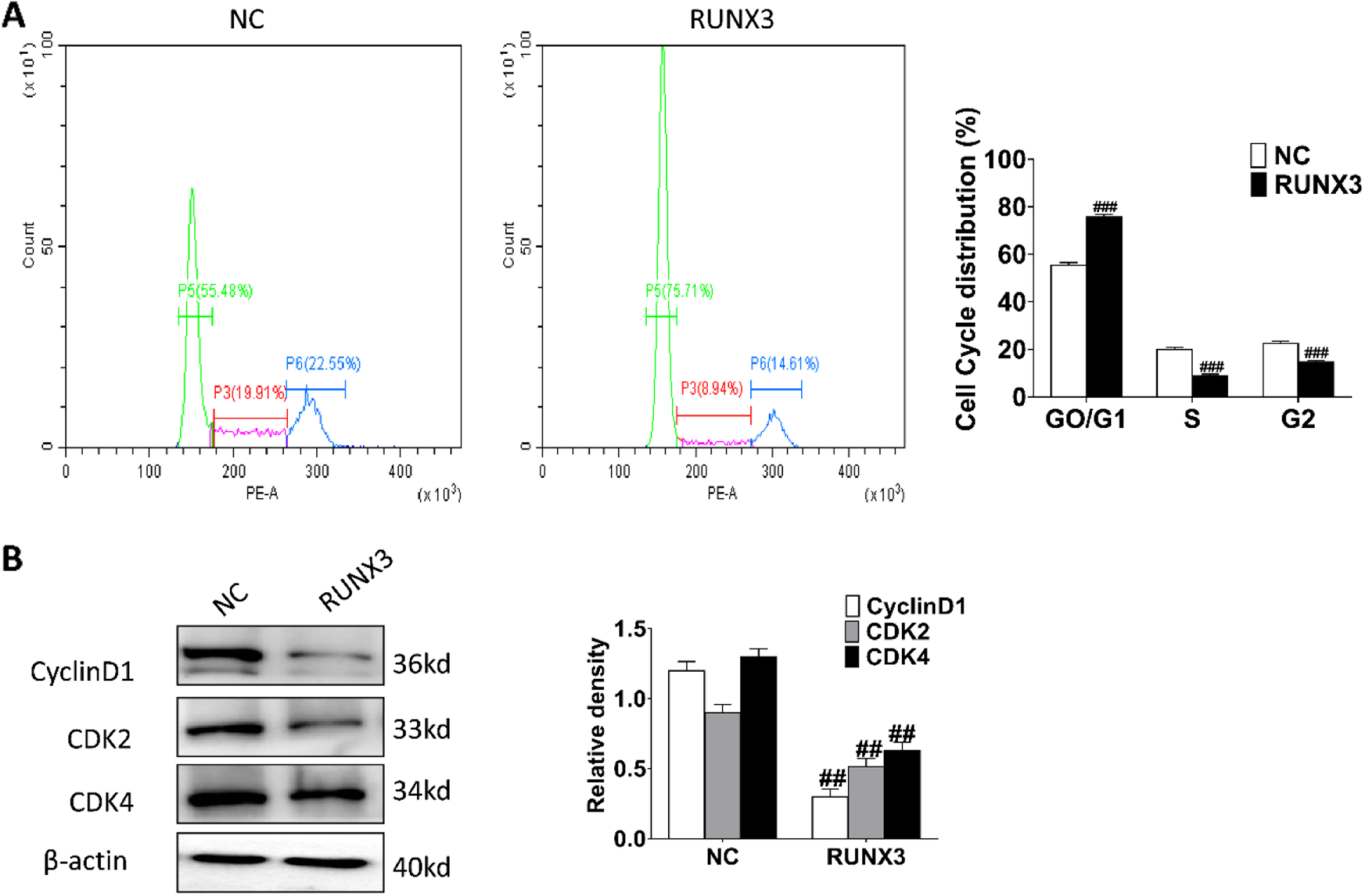
Effect of overexpression of RUNX3 on cell cycle of human keloid fibroblasts. **A** Effect of overexpression of RUNX3 on cell cycle; ###*P* < 0.001 vs NC. **B** Effect of overexpression of RUNX3 on the expression of cycle-related proteins; Original blots are presented in Supplementary Fig. 4. ##*P* < 0.01 vs NC

RUNX3 was over-expressed in keloid fibroblasts (Fig. 4). Cell proliferation was detected with CFSE after overexpression of RUNX3, which showed that RUNX3 overexpression significantly inhibited cell proliferation of keloid fibroblasts (Fig. 5A). Cell scratch assay showed that the migration distance of cells with RUNX3 overexpression was shorter than that of control (Fig. 5B). Analysis of cell cycle showed that cells overexpressing RUNX3 had an increased proportion of G0/G1 (*P* < 0.001) phase and significantly shorter S (*P* < 0.001) and G2/M phases (*P* < 0.001) (Fig. 6A). Examination of cell cycle-related proteins revealed that overexpression of RUNX3 significantly decreased the expression of CDK2 (*P* < 0.01), CDK4 (*P* < 0.01), and CyclinD1 (*P* < 0.01) (Fig. 6B). Thus, RUNX3 may inhibit cell proliferation by regulating the cell cycle.

### Effect of SIRT1 on EZH2 acetylation levels in keloid fibroblasts

**Fig. 7:**
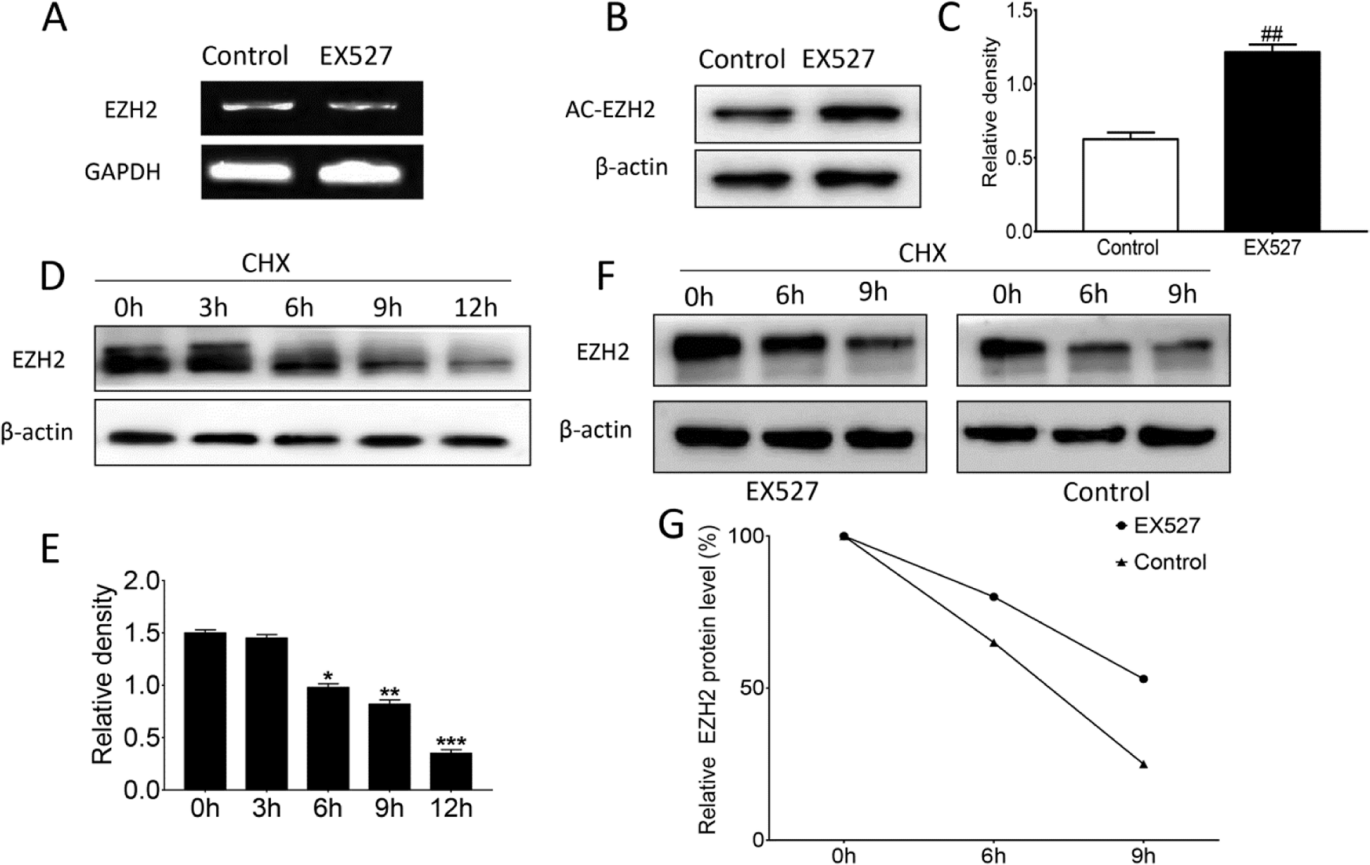
Effect of SIRT1 on EZH2 acetylation levels in keloid fibroblasts. **A** *EZH2* mRNA by RT-PCR. **B-C** Effect of SIRT inhibitor EX527 on EZH2 Acetylation Level. ##*P* < 0.01 vs Control. **D-E** Effect of CHX on protein stability was detected with Western blot. Representative (**D**) and quantitative (**E**) results were shown. *P < 0.05 vs 0 h, **P < 0.01vs 0 h, ***P < 0.001 vs 0 h. **F-G** Expression of protein stability in human keloid fibroblasts treated with SIRT1 inhibitor EX527. Representative (**F**) and quantitative (**G**) results were shown. Original blots/gels are presented in Supplementary Fig. 5 and 6

It is reported that SIRT1 regulates EZH2 activity and stability by acetylating lysine 348 in HEK293T cells [25]. The transcript levels of *EZH2* after treatment of EX527, an SIRT1 inhibitor, were examined by means of RT-PCR, and it was found that SIRT1 inhibition did not have an effect on *EZH2* at the transcript level (Fig. 7A). On the other hand, in addition to changes in transcription level, changes in protein stability can also alter the levels of specific proteins. Thus, we performed protein stability assays. We found that treatment of cells with the SIRT inhibitor EX527 greatly elevated AC-EZH2 levels, illustrating that EX527 could elevate EZH2 acetylation levels (*P* < 0.01) (Fig. 7B and C). To determine the half-life of EZH2 protein, HKF cells were treated with 0.1 μg/mL CHX for various times and Western blot was used to detect protein expression levels. The results revealed that EZH2 protein levels remained stable within 3 h and decreased after 6 h (*P* < 0.05) (Fig. 7D-G). These results indicated that the protein stability of EZH2 increased after inhibition of SIRT1, contributing to the improvement of its protein level.

### Effects of SIRT1/EZH2 Axis cell proliferation

**Fig. 8:**
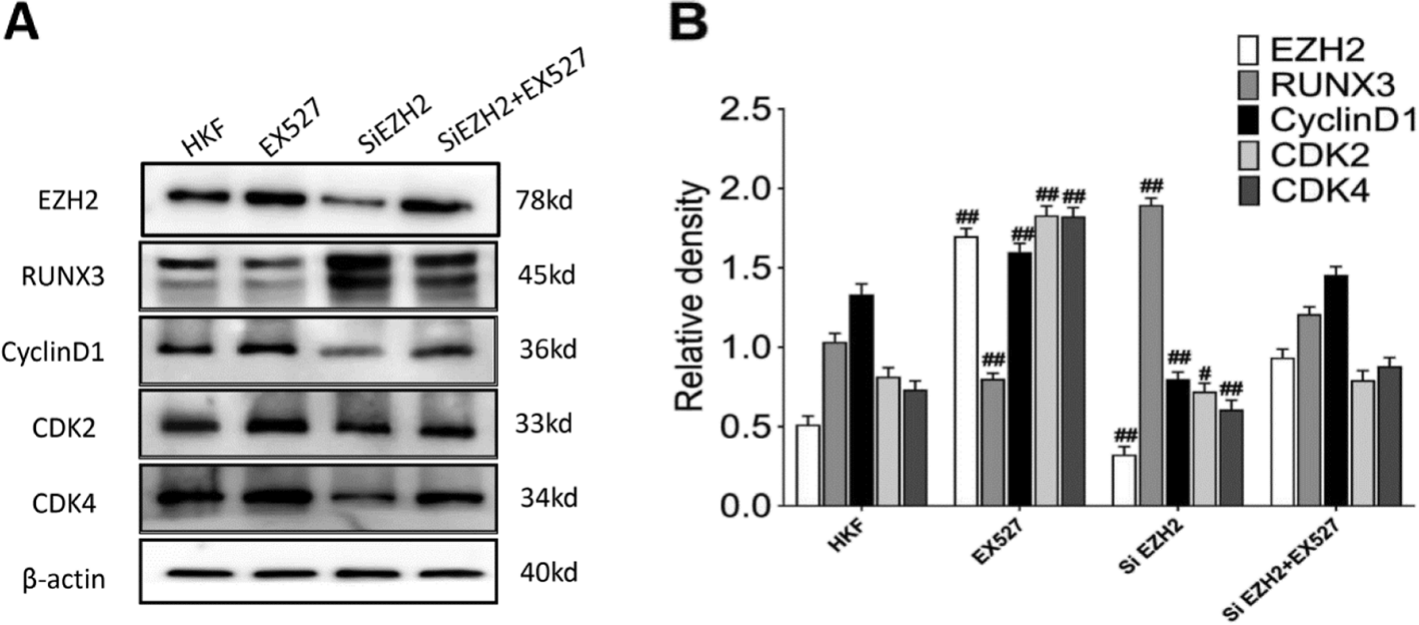
Effects of SIRT1/EZH2 on Cyclins. (**A-B**) Effects of EX527 and knockdown of EZH2 on cyclins, RUNX3, and EZH2. Representative (**A**) and quantitative (**B**) Western blot results were shown. #*P* < 0.05 vs HKF, ##*P* < 0.01 vs HKF. Original blots are presented in Supplementary Fig. 7

**Fig. 9:**
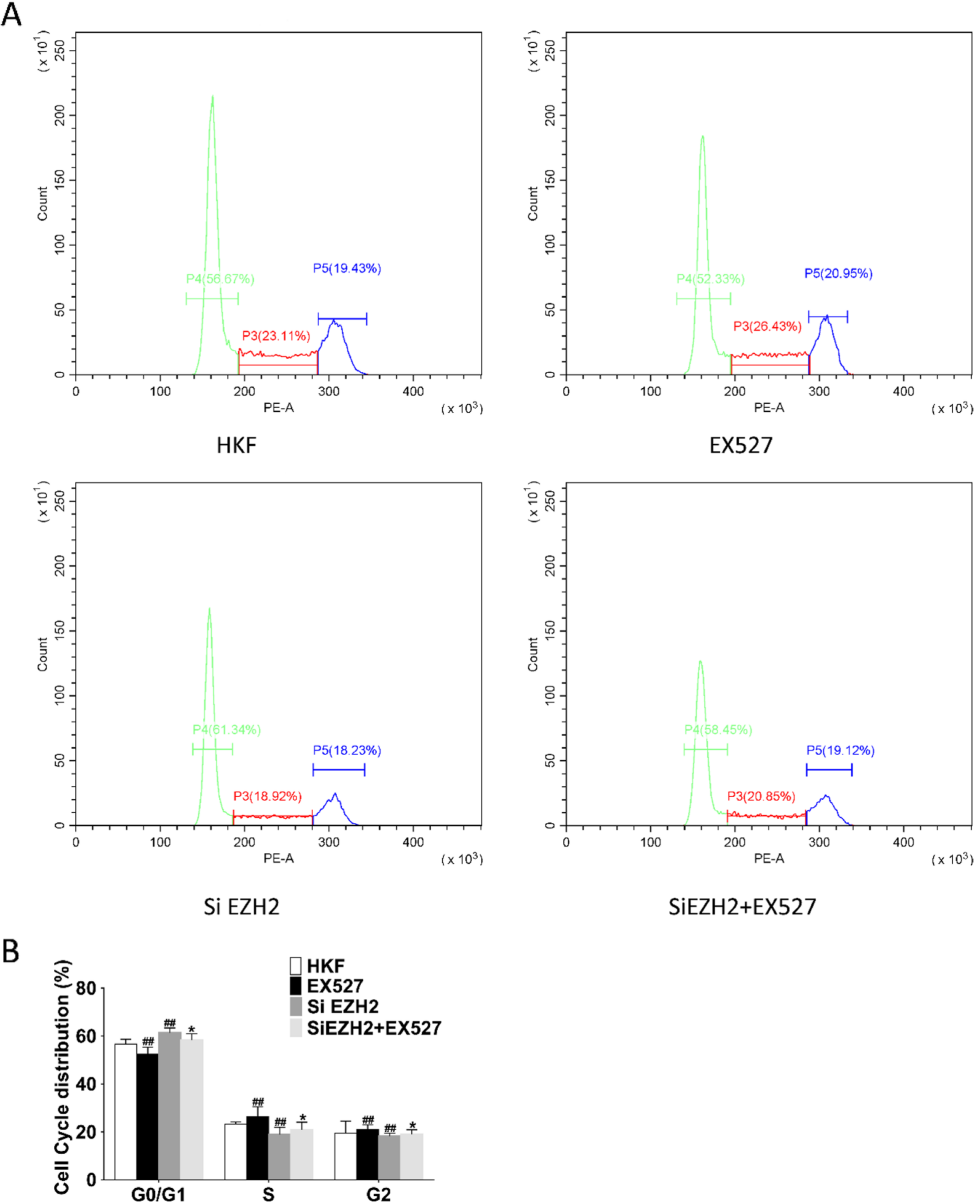
Effects of SIRT1/EZH2 on cell cycle of human keloid fibroblasts. **A** Effect on cell cycle after treatment of EX527 and knockdown of EZH2. **B** Representative and quantitative Western blot results of RUNX3. #*P* < 0.05 vs HKF, ##*P* < 0.01 vs HKF, **P < 0.01 vs EX527

**Fig. 10:**
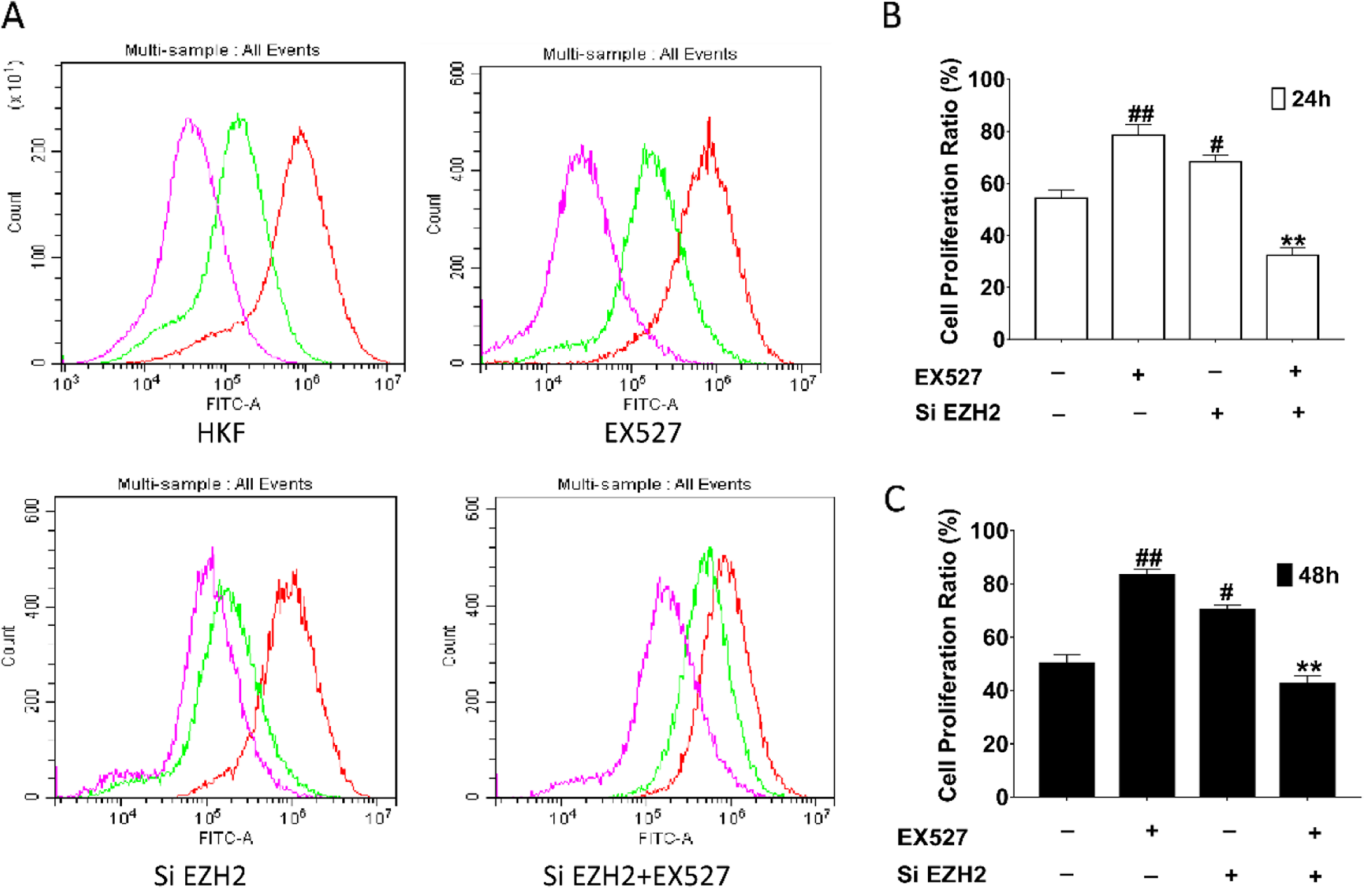
Effects of SIRT1/EZH2 on proliferation of human keloid. Effect on Cell Proliferation after treatment of EX527 and knockdown of EZH2. **A** Green line indicates proliferation at 24 h and red line represent proliferation at 48 h. **B** Cell proliferation ratio at 24 h; (**C**) Cell proliferation ratio at 48 h. #*P* < 0.05 vs HKF, ##*P* < 0.01 vs HKF

To verify the regulation between SIRT1/EZH2 and the effect of SIRT1/EZH2 axis on proliferation, EX527 (an SIRT1 inhibitor) treatment and siEZH2 transfection was performed. Detection of cell cycle-related protein CDK2 (*P* < 0.01), CDK4 (*P* < 0.01) and CyclinD1 (*P* < 0.01) revealed that EX527 and SiEZH2 inhibited their expression, while SiEZH2 + EX527 could promote their protein expression (Figs. 8A and B). The proliferation of keloid fibroblasts treated with EX527 was inhibited, and the S and G2 phases of the cell cycle were shortened according to cell cycle analysis (*P* < 0.01) (Fig. 9A and B), indicating that SIRT1 can regulate the cell cycle, and the same situation was observed after siEZH2 transfection. Interestingly, administration of EX527 after transfection with EZH2 revealed recovery of cell proliferation, prolonged S phase of cells, and shortened G0/G1 phase (*P* < 0.05) (Fig. 10A-C). In summary, SIRT1 could regulate EZH2 by deacetylating and attenuating its expression.

## Discussion

The current treatment for keloids includes traditional surgical excision, cryotherapy, cryotherapy in combination with other methods such as intralesional steroid injection, external beam therapy, brachytherapy, and laser therapy [26–28]. Keloid tissue is characterized by excessive accumulation of extracellular matrix collagen in the dermis and subcutaneous tissue. Study has shown that Pa-PDT may be a potential treatment modality for keloids, while RUNX3 may improve the sensitivity to PaPDT in keloid fibroblast KFs and thus the effect on the scar airway [13]. It has been shown that p300-mediated acetylation of RUNX3 induced by TGF-β signaling inhibits the degradation of RUNX3 [29]. In addition, phosphorylation of RUNX3 by Src reduces the nuclear import of RUNX3, thereby reducing its transactivation activity [30]. It is shown that G9a may directly regulate RUNX3 to regulate the tumor suppressor function of RUNX3 by methylation [31].

In this study, we first found that in keloid tissues, the expression level of RUNX3 was significantly lower than that in normal tissues. Considering that RUNX3 may act at tumor suppressor gene in keloids, we analyzed the correlation of SIRT1, EZH2, and RUNX3 expression in keloid tissues and found that there were negative regulatory relationships between SIRT1 and EZH2, and between EZH2 and RUNX3. SIRT1 and RUNX3 were positively correlated. Further comparison between keloid fibroblasts and normal fibroblasts revealed that the expression of abnormally proliferating HKF was similar to that in keloid tissues, and the abnormal proliferation of keloid fibroblasts was significantly inhibited after overexpression of RUNX3 in keloid fibroblasts. RUNX3 plays an inhibitory role as a tumor suppressor gene in a variety of cancers. For example, RUNX3 is considered to act as a new tumor suppressor gene in the breast for degradation by targeting the estrogen receptor [32], and different post-translational modifications affect the tumor suppressor function of RUNX3, such as stability, subcellular localization, and transactivation activity [33]. In this study, we found that the overexpression of RUNX3 in keloid fibroblasts helped to inhibit cell proliferation, and the detection of cell cycle and cell cycle-related proteins revealed that the effect of RUNX3 on cell proliferation may be related to its regulation on cell cycle. The proportion of G0/G1 phase increased, and that of S phase and G2 phase shortened in keloid fibroblasts overexpressing RUNX3. CDK1, as a key protein promoting cell division and proliferation, binds to CDK4 to form a complex in the cell cycle and shortens the cell cycle, while CDK2 also plays a role as a key protein in the cell cycle by phosphorylating Rb protein to activate E2F transcription factor on S phase gene expression and ensure that cells smoothly enter S phase from G1 phase. The effect of overexpression of RUNX3 on CDK2/CDK4 as well as CyclinD1 indicates that RUNX3 can promote G1/S phase progression of the cell cycle.

SIRT1 has been shown to bind to and deacetate many transcription factors such as p53 and FOXO3a, and the interaction of SIRT1 with each transcription factor, among others, suggests that SIRT1 plays a role in cell survival, metabolism, DNA repair and tumorigenesis [34]. The pRB-E2F pathway is also a major regulatory pathway regulating EZH2 expression [31], and SIRT1 may regulate EZH2 expression at the transcriptional level [35]. SIRT1 has been shown to be present in the promoters of some tumor suppressor genes, leading to hyperacetylation of H4K16, which in turn induces the expression of these genes [35]. In this study, we used the SIRT1 inhibitor EX527 and examined EZH2 acetylation levels, and inhibition of SIRT1 significantly increased EZH2 acetylation levels. Stability testing of EZH2 revealed that SIRT1 had an effect on the protein degradation of EZH2 from 6 h. Inhibition of SIRT1 increased SIRT1 acetylation levels and decreased EZH2 degradation after 6—9 h. However, it had no significant effect on the expression of *EZH2* at the transcriptional level. In this study, we examined RUNX3 expression by knocking down EZH2 and found that inhibiting EZH2 promoted RUNX3 expression, inhibited cell proliferation, and regulated the cell cycle. Similarly, incubation of cells with SIRT1 inhibitor EX527 revealed that inhibition of SIRT1 inhibited the expression level of RUNX3 in addition to increasing EZH2. SIRT1 inhibitor could promote S phase prolongation and increase the expression of cyclin in proliferating cells after EX527 use. EZH2-transfected cells were detected for protein and proliferation after using EX527 for 24 h, and it was found that EX527 could reverse the effect of EZH2.

This study has some limitations. For example, the sample size of participants was small.

## Conclusions

In summary, our study shows that SIRT1 could promote proliferation by reducing RUNX3 expression in keloids through deacetylation of EZH2. SIRT1/EZH2/RUNX3 axis may be important for regulating abnormal proliferation in keloids.

## Methods

### Study subjects

Keloid tissues and surrounding normal skin tissues were collected from 5 patients with keloid. The keloids were removed according to previous description [36]. This study was reviewed and approved by the ethics committee of Yanbian Hospital (Approval No: ybyy-2020–03,010). All methods were performed in accordance with the relevant guidelines and regulations. Patients or their legal guardians were informed and consented to the study before sample collection and signed the informed consent.

### Cell culture

Human keloid fibroblasts (HKF) were purchased from Hunan Fenghui Biotechnology Co., Ltd. (Changsha, China). Human fibroblasts were from Yanbian Hospital. They were cultured in DMEM/F12 culture medium containing 10% FBS and 100 U/ml penicillin and 100 μg/ml streptomycin at 37° C with 5% CO_2_.

### HE staining

Keloid tissues and surrounding normal skin tissues were fixed in 10% formaldehyde for more than 72 h, washed overnight with running water, embedded in paraffin after gradient dehydration, and cut into 4 μm sections. The sections were subjected to hematoxylin staining for 5 min and eosin staining for 1 min. The pathological changes were observed under microscope.

### Cell transfection

Cells in logarithmic growth phase were transfected with siNC, siRUNX3, siNC and siEZH2 for 24 h by using Lipofectamine 3000. At 24 h after transfection, SiEZH2 + EX527 groups were treated with EX527 (10 μM; MedChemExpress) for 24 h.

### RT-PCR

Cellular RNA was extracted with RNA simple Total RNA Kit (DP419; TIANGEN BIOTECH CO., Ltd, Beijing, China). Reverse transcription was performed. The primer sequences were: *EZH*2, forward: 5 ‘-GCCAGACTGGGAAGAAATCTG-3’, reverse: 5 ‘-TGTTGGAAAATCCAAGTCA-3’; *RUNX3*, forward: Reverse TTTCACCCTGACCATCACTGTG, reverse: GCATCCCACTGGTGAAGGTTG; and, *GAPDH*, forward: 5 ‘-GGTGAAGGTCGGAGTCAACGGA-3’, forward: 5 ‘-GAGGGATCTCGCTCCTGGAAGA-3’. The FastKing One Step RT-PCR Kit (TIANGEN BIOTECH CO., Ltd, Beijing, China) was used. The RT-PCR system (total volume 25 µl) included 2 × FastKing One Step RT-PCR Master Mix 12.5 μl, 25 × RT-PCR Enzyme Mix1 µl, forward primer (10 µM) 0.6 µl, reverse primer (10 µM) 0.6 µl, RNA template 1 µl, and RNase-FreeddH_2_O 9.3 µl. The RT-PCR procedure included 42° C for 30 min, 95° C for 3 min; 35 cycles of 94° C for 30 s, 55° C for 20 s, and 72℃ for 30 s; and 72° C for 5 min. The PCR products were analyzed by agarose gel electrophoresis.

### Immunoprecipitation

Proteins were extracted from HKF with protein extraction kit. Freshly extracted protein sample (200 μl) was incubated with 2 μl of acetyl-lysine antibody at 4° C overnight. Then, 40 μl of fully resuspended ProteinA + G Agrarose beads (Beyotime Biotechnology) was added and incubated at 4° C for 3 h, followed by centrifugation at 3000 r/min for 5 min at 4° C. The pellet was collected, washed five times with precooled PBS, and subjected to Western blot analysis of acetylated EZH2.

### Protein stability testing

To determine the half-life of EZH2 protein, HKF cells were treated with 0.1 μg/mL cyclohexylamine (CHX) (Sigma-Aldrich) for 0 h, 3 h, 6 h, 9 h and 12 h, and EZH2 protein levels were measured by Western blot. Based on the observations in this experiment, three time points were selected to test cells after EX527 treatment with 0.1 μg/mL CHX. The treated cells were then washed three times with cold PBS to remove CHX, followed by extraction of whole cell lysate and Western blot.

### Scratch test

Keloid fibroblasts were cultured in 12-well plates, and the transfection efficiency was observed by Cytation5 after 24 h of RUNX3 transfection. Scratches were made on the plate with a tip. After washing three times with PBS, the cell culture was continued with serum-free culture medium. The cells were photographed at 0, 12, and 24 h after scratch.

### CFSE proliferation assay

Cells were seeded in a 6-well plate (5 × 10^4^cells/well), washed with 1 × PBS, and incubated in CFSE working solution (1 μM) at 37° C for 20 min in the dark. The serum-free culture medium was replaced with complete culture medium to continue the culture. After culture for 12 h and 24 h, the cells were collected and detected on the flow cytometer at 530 nm.

### Cell cycle assay

Cells were collected, washed with PBS and incubated with precooled 70% ethanol for 4 h at 4 °C. After washing, PI and RNaseA were added, respectively, and incubated for more than 30 min at 4 °C in the dark. Cell cycle was detected on the flow cytometer (Beckman Coulter Biotechnology (Suzhou) Co, Ltd).

### Western blot

Keloid tissues and its surrounding normal skin tissues were homogenized, lysed in RIPA for 30 min, and centrifuged at 12,000 r/min for 5 min. The supernatant was collected and the protein concentration was determined with BCA method. Similarly, proteins were also extracted from cells. After SDS-PAGE electrophoresis, the proteins were transferred to PVDF membranes. The membrane was blocked with 5% skimmed milk for more than 2 h, and washed with TBST for 5 min/time for 3 times. Then, the diluted primary antibodies against RUNX3 (1:2000; Abcam, USA), EZH2 (1:2000; Abcam, USA), AC-EZH2 (1:2000; Abcam, USA), SIRT1 (1:2000; Abcam, USA), CyclinD1 (1:2000; Abcam, USA), CDK2 (1:2000; Abcam, USA), CDK4 (1:2000; Abcam, USA), and β-actin (1:2000; Abcam, USA) were added and incubated overnight at 4° C. After incubation with goat anti-rabbit/goat anti-mouse secondary antibody (1:5000; Abcam, USA) for 70 min at room temperature, the membrane was subjected to ECL luminescence color development.

### Flow cytometry

The skin tissues were homogenized and filtered to prepare a single cell suspension. After washing and centrifugation, the cells were fixed with 4% paraformaldehyde for 10 min. After washing again, the cells were incubated with 10% methanol for 10 min, and then with 5% BSA for 1 h. After that, the antibodies against Vimentin (1:250; Abcam, USA) and RUNX3 (1:500; Abcam, USA) were added and incubated for 1 h. Following washing, the secondary antibody (1:2000; Abcam, USA) was added for 1 h incubation. Finally, the cells were detected on the flow cytometer (Beckman Coulter Biotechnology (Suzhou) Co, Ltd).

### Statistical methods

Statistical analysis was performed with SPSS version 13.0 (SPSS Inc., Chicago, IL), and all quantitative data are presented as the mean ± standard deviation. Differences between two groups were compared using Student’s t-test when they had a normal distribution. A one-way analysis of variance (ANOVA) was used to compare data among groups when they had a normal distribution and homogeneous variances. A p value less than 0.05 was considered statistically significant.

## Supplementary Information

**Additional file 1:** **Supplementary Figure 1** for Figure 2A (SIRT1), 2B (EZH2) and 2C (RUNX3) expression in keloid and normal tissue: this figure is full image of western blots combines with marker. **Supplementary Figure 2** for Figure 3A (SIRT1, EZH2 and RUNX3) expression in Human Keloid fibroblast and Human fibroblasts: this figure is full image of western blots combines with marker. **Supplementary Figure 3** for Figure 4 (RUNX3 and β-actin) expression after Transfection: this figure is full image of western blots combines with marker. **Supplementary Figure4 **for Figure 6B (CyclinD1, CDK2, CDK4, and β-actin) expression after Transfection: this figure is full image of western blots combines with marker. **Supplementary Figure5** for Figure 7A: expression of *EZH2 *mRNA by RT-PCR and 7B: Effect of SIRT inhibitor EX527 on EZH2 Acetylation Level. **Supplementary Figure6** for Figure 7D-F Effect of CHX on EZH2 protein stability and Expression of EZH2 protein stability in human keloid fibroblasts treated with SIRT1 inhibitor EX527: this figure is full image of western blots combines with marker. **Supplementary Figure 7** for Figure 8A (EZH2, RUNX3, CyclinD1, CDK2, CDK4, and β-actin) after treatment of EX527 and knockdown of EZH2: this figure is full image of western blots combines with marker.

## Data Availability

The datasets generated during and/or analysed during the current study are available from the corresponding author on reasonable request.

## Abbreviations

SIRT1: Sirtuin 1
EZH2: Zeste Homolog 2
RUNX3: Runt-related transcription factor 3
TGF-β: Transforming growth factor beta
H3K27me3: Trimethylation of Lys-27 in histone 3
HKF: Human keloid fibroblasts
CHX: Cyclohexylamine
ANOVA: Analysis of variance
