# Supplementary material for: RUNX3 mediates keloid fibroblast proliferation through deacetylation of EZH2 by SIRT1
Source: BMC Mol Cell Biol. 2022 Dec 7;23:52. doi: 10.1186/s12860-022-00451-4 (PMC9730640; doi:10.1186/s12860-022-00451-4)

Supplementary Figure1 for Figure 2A (SIRT1), 2B (EZH2) and 2C (RUNX3) expression in keloid and normal tissue: this figure is full image of western blots combines with marker

2A: SIRT1

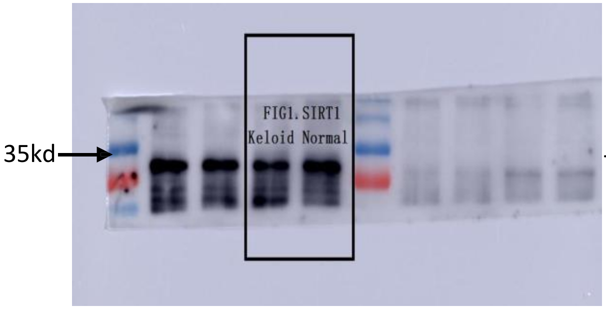

2B: EZH2

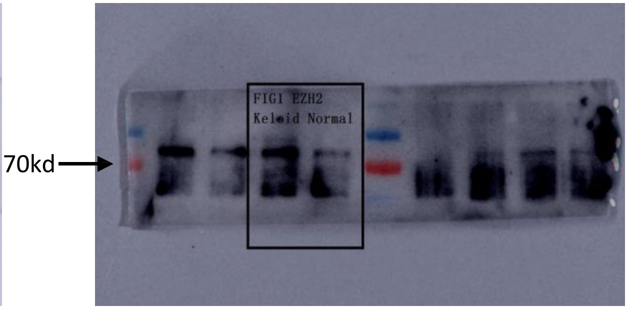

2C: RUNX3

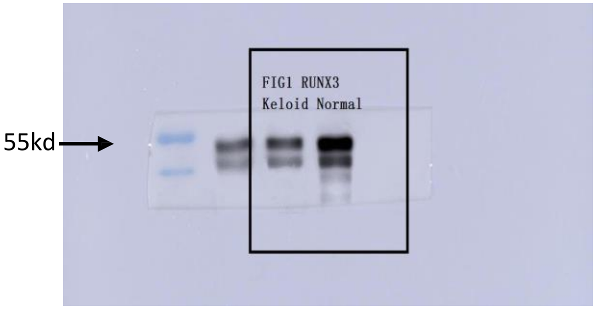

2A: β-actin

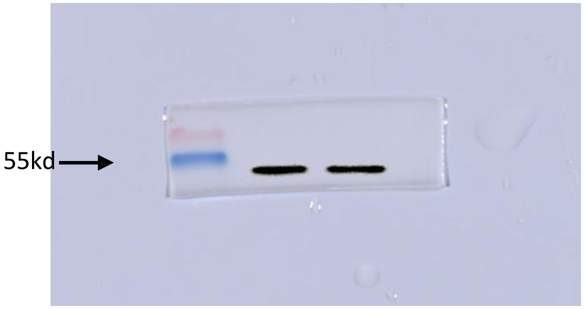

2B: β-actin

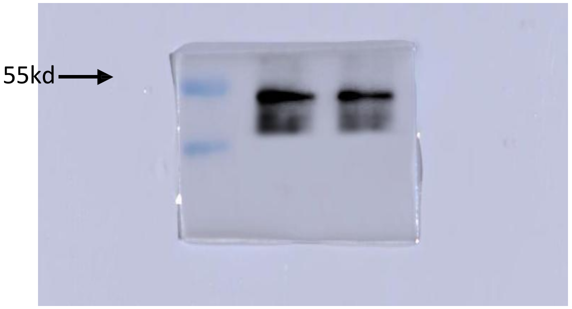

2C: β-actin

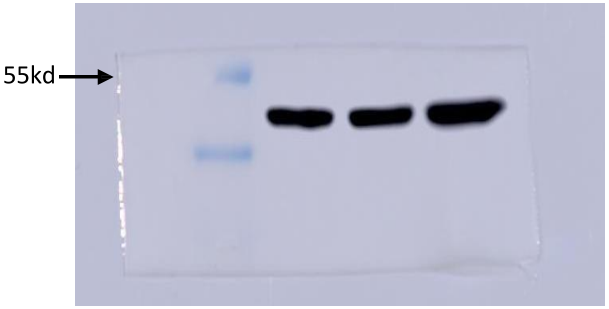

Supplementary Figure2 for Figure 3A (SIRT1, EZH2 and RUNX3) expression in Human Keloid fibroblast and Human fibroblasts: this figure is full image of western blots combines with marker

3A: SIRT1

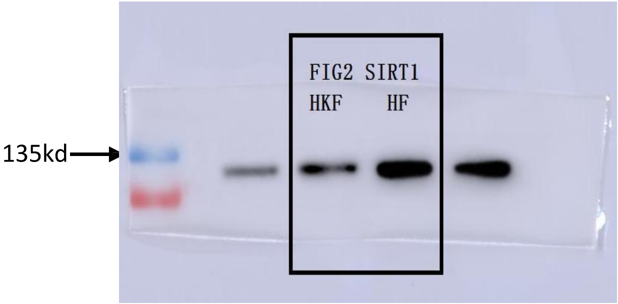

3A: EZH2

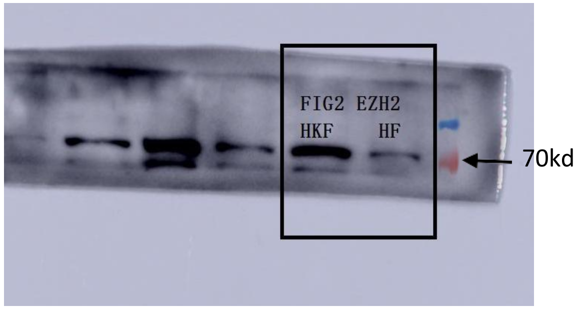

3A: RUNX3

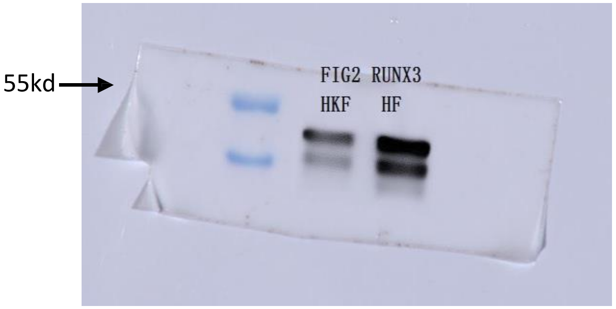

3A: β-actin

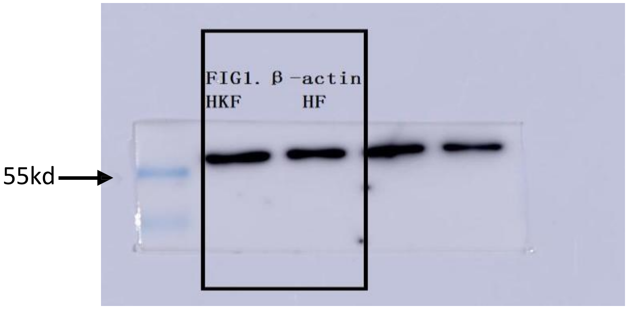

Supplementary Figure 3 for Figure 4 (RUNX3 and β-actin) expression after Transfection: this figure is full image of western blots combines with marker

Fig. 4: RUNX3

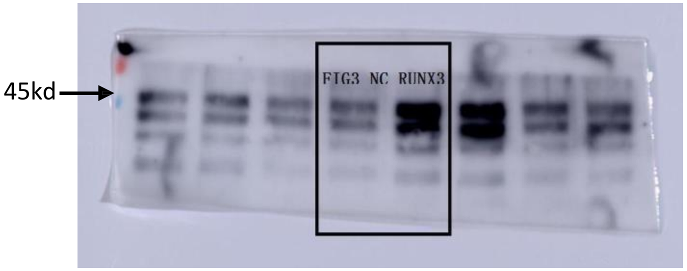

Fig. 4: β-actin

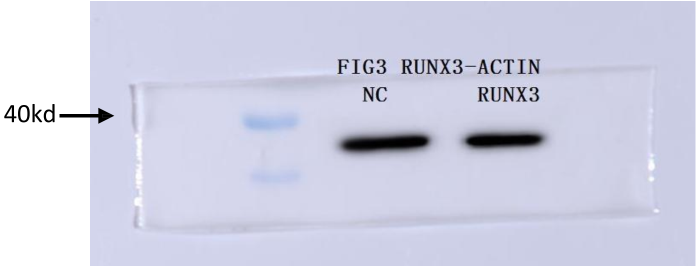

Supplementary Figure4 for Figure 6B (CyclinD1, CDK2, CDK4, and β-actin) expression after Transfection: this figure is full image of western blots combines with marker

6B: CyclinD1

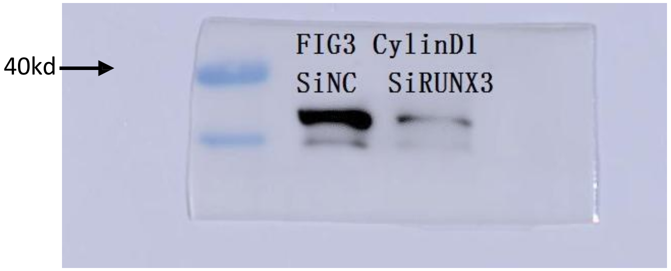

6B: CDK2

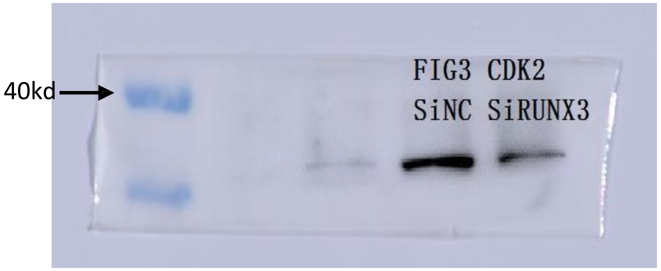

6B: CDK4

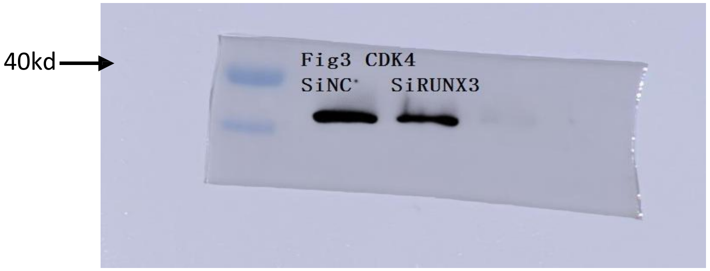

6B: β-actin

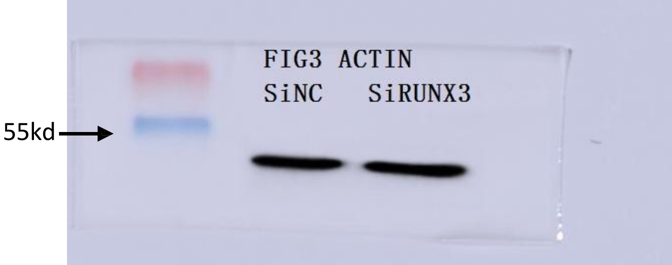

Supplementary Figure5 for Figure 7A: expression of *EZH2* mRNA by RT-PCR and 7B: Effect of SIRT inhibitor EX527 on EZH2 Acetylation Level

FIG 7A

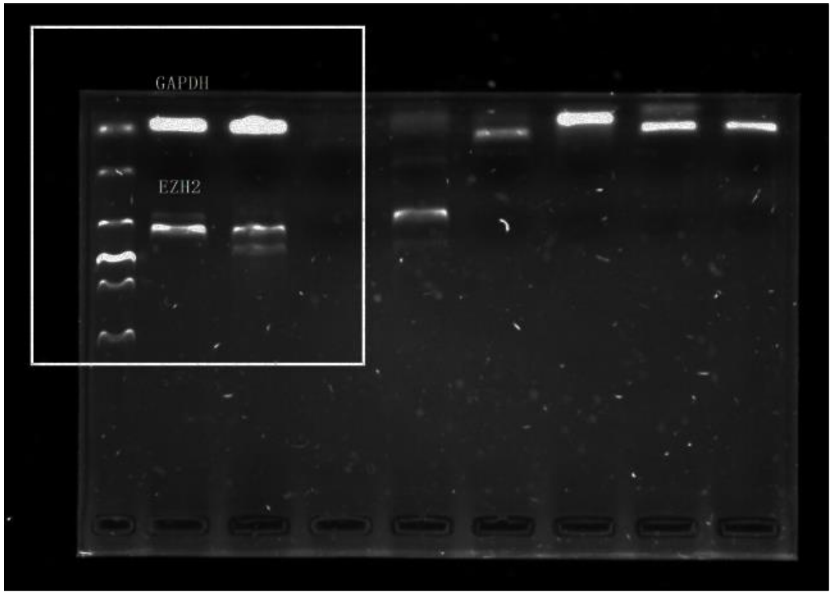

FIG 7B AC-EZH2

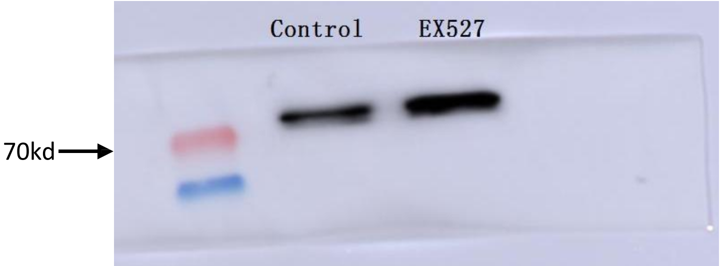

FIG 7B β-actin

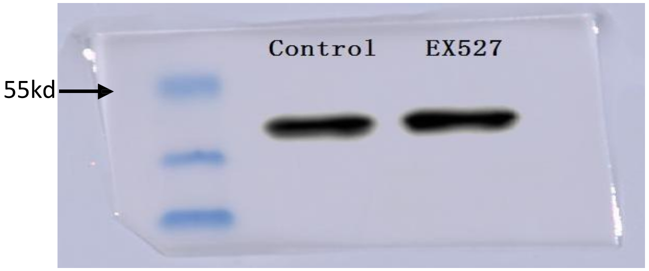

Supplementary Figure6 for Figure 7D-F Effect of CHX on EZH2 protein stability and Expression of EZH2 protein stability in human keloid fibroblasts treated with SIRT1 inhibitor EX527: this figure is full image of western blots combines with marker

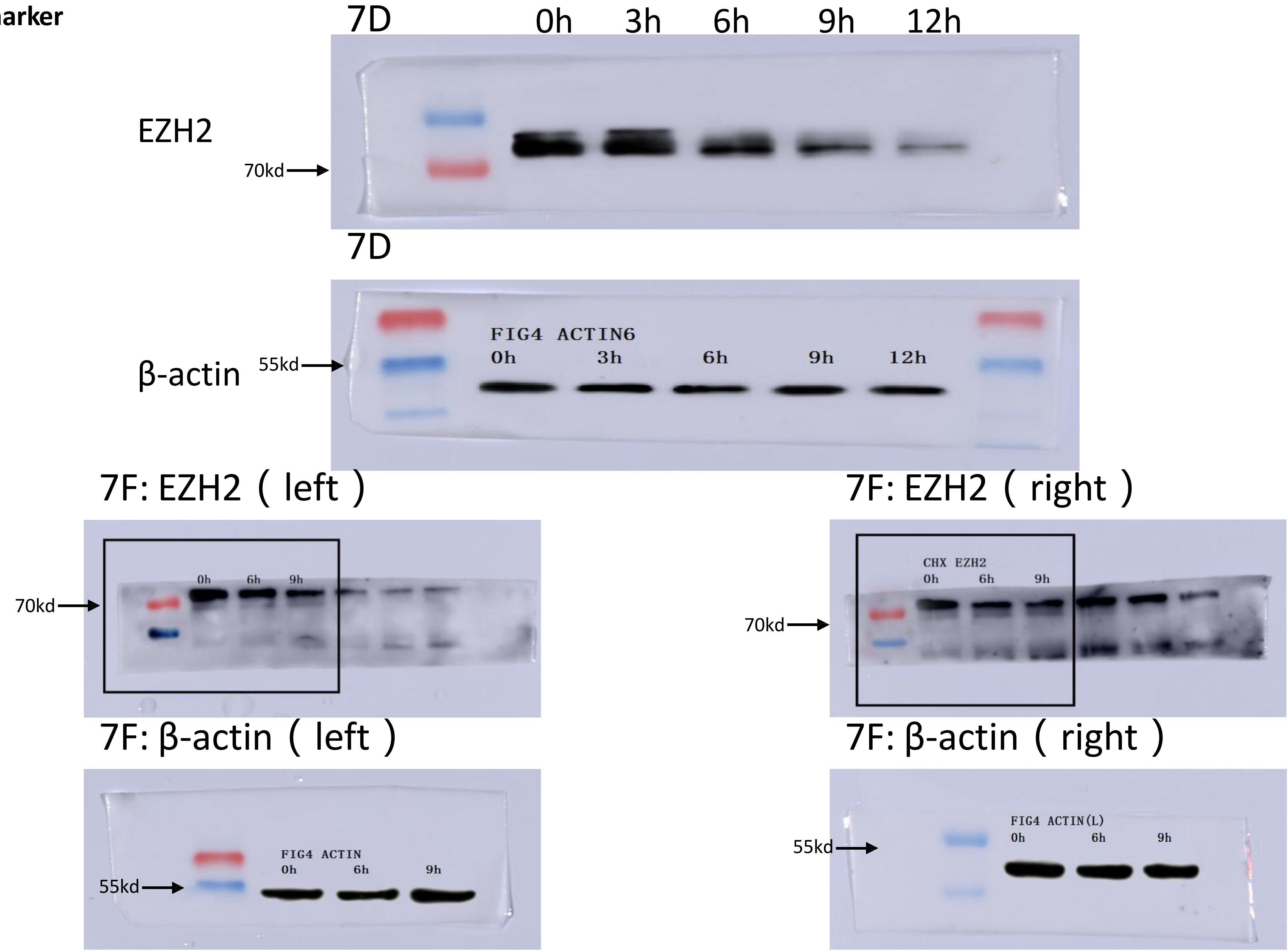

Supplementary Figure 7 for Figure 8A (EZH2, RUNX3, CyclinD1, CDK2, CDK4, and β-actin) after treatment of EX527 and knockdown of EZH2: this figure is full image of western blots combines with marker

8A: EZH2

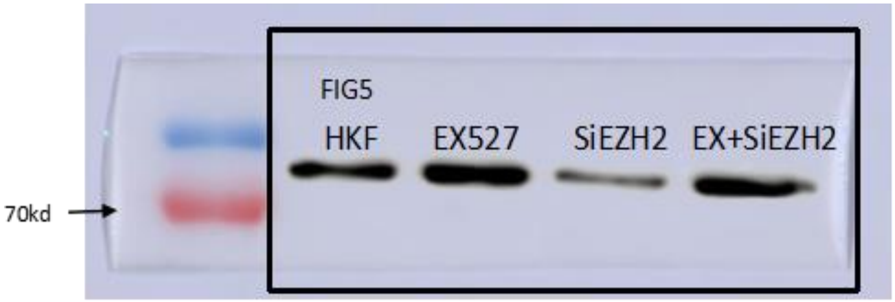

8A: RUNX3

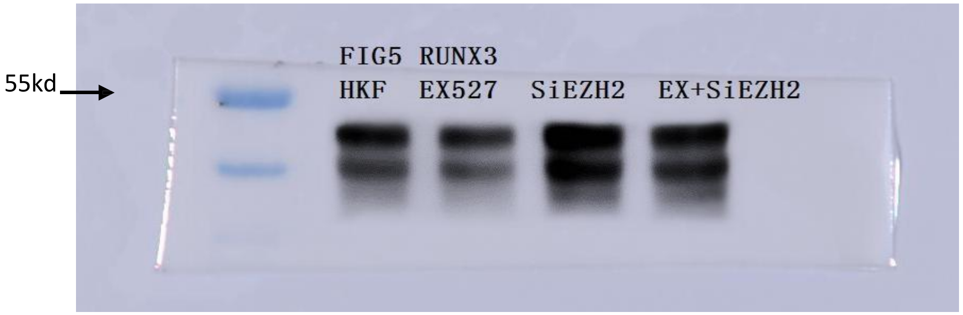

8A: CyclinD1

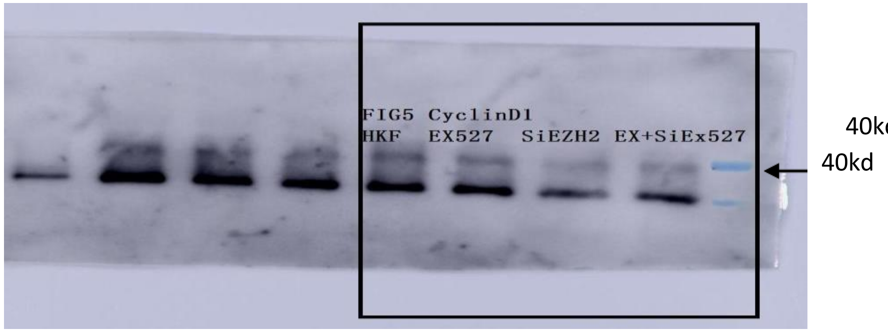

8A: CDK2

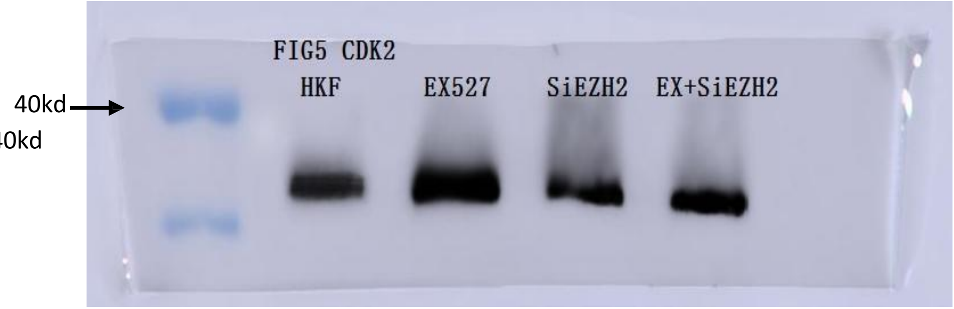

8A: CDK4

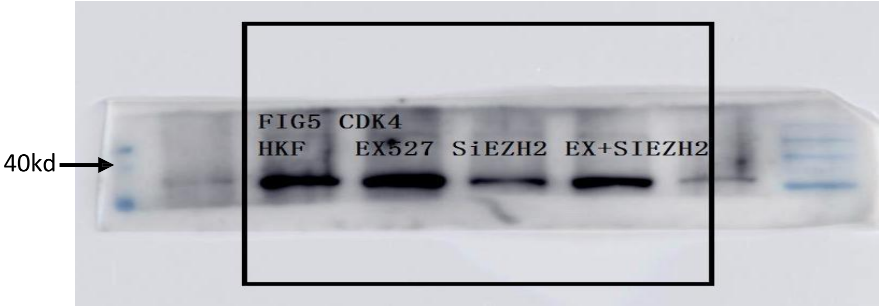

8A: β-actin

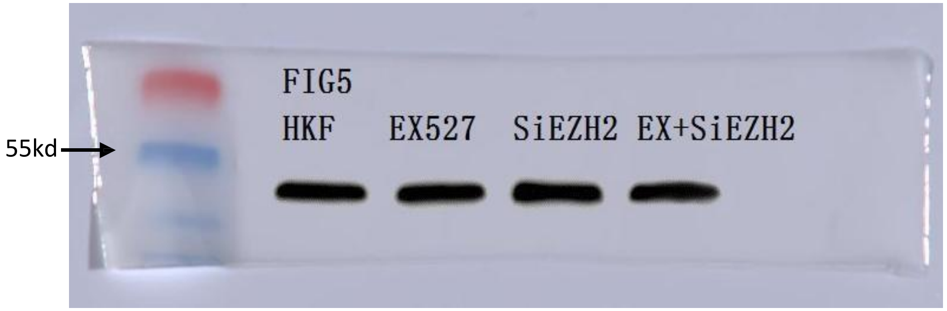

Supplement: Supplementary file 1 — Additional file 1: Supplementary Figure 1 for Figure 2A (SIRT1), 2B (EZH2) and 2C (RUNX3) expression in keloid and normal tissue: this figure is full image of western blots combines with marker. Supplementary Figure 2 for Figure 3A (SIRT1, EZH2 and RUNX3) expression in Human Keloid fibroblast and Human fibroblasts: this figure is full image of western blots combines with marker. Supplementary Figure 3 for Figure 4 (RUNX3 and β-actin) expression after Transfection: this figure is full image of western blots combines with marker. Supplementary Figure4 for Figure 6B (CyclinD1, CDK2, CDK4, and β-actin) expression after Transfection: this figure is full image of western blots combines with marker. Supplementary Figure5 for Figure 7A: expression of EZH2 mRNA by RT-PCR and 7B: Effect of SIRT inhibitor EX527 on EZH2 Acetylation Level. Supplementary Figure6 for Figure 7D-F Effect of CHX on EZH2 protein stability and Expression of EZH2 protein stability in human keloid fibroblasts treated with SIRT1 inhibitor EX527: this figure is full image of western blots combines with marker. Supplementary Figure 7 for Figure 8A (EZH2, RUNX3, CyclinD1, CDK2, CDK4, and β-actin) after treatment of EX527 and knockdown of EZH2: this figure is full image of western blots combines with marker. [file 12860_2022_451_MOESM1_ESM.pdf]
